# Collaboration and co-design between research experts and experts with lived experience in the TRUSTING project for AI-based relapse risk assessment for individuals with psychosis

**DOI:** 10.1192/j.eurpsy.2026.10521

**Published:** 2026-07-31

**Authors:** S. A. Just, E. Van der Eycken, E. Tedeschi, M. Hussain, B. Elvevåg

**Affiliations:** 1 Department of Clinical Medicine, UiT - The Arctic University of Norway, Tromsø, Norway; 2 Charité – Universitätsmedizin Berlin, Berlin, Germany; 3 Global Alliance of Mental Illness Advocacy Networks-Europe (GAMIAN-Europe), Brussels, Belgium; 4 Department of Computer Science, UiT - The Arctic University of Norway, Tromsø, Norway

## Abstract

**Introduction:**

Healthcare systems often struggle to adequately address the needs of people with mental health conditions, leading to experiences of stigmatization, discrimination, and inadequate care. These shortcomings can deter individuals from seeking or engaging with treatment, particularly when interventions are developed without meaningful user involvement. Digital tools must be held to an especially high standard in this context, as they raise additional concerns around data privacy, security, and misuse of sensitive data. Still, digital mental health holds significant therapeutic potential, and research shows that individuals with mental health conditions view AI-based tools favorably when privacy concerns are adequately addressed.

**Objectives:**

The TRUSTING project – a prospective multicenter study developing AI-based relapse risk assessment through language analysis in individuals with psychosis – addresses these challenges by including the perspectives of experts with lived experience.

**Methods:**

Crucial technical components such as the TRUSTING app for data collection were co-designed in the project. A dedicated advisory user board, established by GAMIAN-Europe, provided continuous input on technical development to UiT – The Arctic University of Norway. In addition, several rounds of surveys among people with lived experience asked about their opinion on digital health and mental health care.

**Results:**

In our presentation, we will discuss the collaborative design process, analyzing both successful strategies and challenges. Moreover, we will present preliminary findings from the surveys, highlighting service user needs and key factors influencing engagement with digital mental health solutions.

**Conclusions:**

The TRUSTING project illustrates how collaborative design with service users can address barriers in digital mental health and enhance the relevance of emerging AI-based tools.

**Disclosure of Interest:**

None Declared

